# Influence of glycemic index and glycemic load of the diet on the risk of overweight and adiposity in childhood

**DOI:** 10.1016/j.rppede.2015.12.009

**Published:** 2016

**Authors:** Kellen Cristine Silva, Luciana Neri Nobre, Sofia Emanuelle de Castro Ferreira Vicente, Lidiane Lopes Moreira, Angelina do Carmo Lessa, Joel Alves Lamounier

**Affiliations:** a Universidade Federal do Tocantins, Palmas, TO, Brazil; b Universidade Federal dos Vales do Jequitinhonha e Mucuri, Diamantina, MG, Brazil; c Universidade Federal de São Paulo, São Paulo, SP, Brazil; d Universidade Federal de Minas Gerais, Belo Horizonte, MG, Brazil; e Universidade Federal de São João Del Rey, São João Del Rey, MG, Brazil

**Keywords:** Glycemic index, Overweight, Children, Adiposity, Carbohydrate

## Abstract

**Objective:**

To investigate the association between the glycemic index and the glycemic load of the diet with the risk of overweight and high adiposity in children with 5 years of age.

**Methods:**

Cross-sectional study nested in a cohort of 232 children born and living in Diamantina (MG, Brazil). Parents and/or guardians provided the food intake data, using a semiquantitative food frequency questionnaire, past history and socioeconomic conditions. Anthropometric and fatness data were collected from the children. The dietary glycemic index and the glycemic load were calculated from the food intake. The glycemic index and glycemic load effect on overweight and adiposity in children was assessed by the Poisson regression (*p*<0.05).

**Results:**

The prevalence of overweight by body mass index was 17.3%, and high adiposity was observed in 3.4% and 6.9% by triceps skinfold and subscapular skinfold, respectively. No difference was reported between the mean body mass index, triceps skinfold and subscapular skinfold according to the glycemic index and glycemic load tertiles; however, the overweight group presented a higher carbohydrate intake (*p*=0.04). No association was found between glycemic index and glycemic load with overweight and adiposity among the children assessed.

**Conclusions:**

The glycemic index and glycemic load of the diet were not identified as risk factors for overweight and adiposity in this cross-sectional study.

## Resumo

**Objetivo:**

Investigar a associação entre o índice glicêmico e a carga glicêmica da dieta sobre o risco de sobrepeso e adiposidade em crianças de cinco anos.

**Métodos:**

Estudo transversal aninhado a uma coorte de 232 crianças nascidas e residentes em Diamantina (MG, Brasil). Os pais e/ou responsáveis forneceram os dados de consumo alimentar, usando num questionário semiquantitativo de frequência alimentar, histórico do paciente e condições socioeconômicas. Os dados antropométricos e a gordura corporal foram coletados das crianças. O índice glicêmico da dieta e a carga glicêmica foram calculados a partir da ingestão de alimentos. O efeito do índice glicêmico e da carga glicêmica no sobrepeso e na adiposidade das crianças foi avaliado por meio da regressão de Poisson (*p*<0,05).

**Resultados:**

A prevalência de sobrepeso pelo índice de massa corporal foi de 17,3% e adiposidade elevada foi observada em 3,4% e 6,9% por meio da prega cutânea do tríceps e da prega cutânea subescapular, respectivamente. Não houve diferença entre a média de índice de massa corporal, prega cutânea do tríceps e prega cutânea subescapular de acordo com os tercis de índice glicêmico e carga glicêmica; no entanto, o grupo com sobrepeso apresentou maior ingestão de carboidratos (*p*=0,04). Não foi encontrada associação entre índice glicêmico e carga glicêmica com sobrepeso e adiposidade entre as crianças avaliadas.

**Conclusões:**

O índice glicêmico e carga glicêmica da dieta não foram identificados como fatores de risco para sobrepeso e adiposidade neste estudo transversal.

## Introduction

The Brazilian population, particularly children, has experienced a nutritional transition characterized by a decrease in malnutrition and a corresponding increase in the prevalence of overweight and obesity.^1^^,^ 2 Corresponding to this process, a drop in the consumption of cereals, beans and tubers was observed with a subsequent increased intake of processed foods, like sandwiches, cookies, snacks and sweets.^2^

This change in the consumption pattern predisposes the intake of a high-refined carbohydrate diet, characterized by a high Glycemic Index (GI) and a high Glycemic Load (GL). The GI is defined as the incremental area under the glycemic response curve post consumption of 25 or 50g of the carbohydrates available in a food. This parameter is expressed as a percentage relative to the glycemic response of a reference food (glucose or white bread) which, thus, reflects the quality of the carbohydrate.^3^ The GL is the product of the GI of the food and its available carbohydrate content; therefore, it represents both the quality and quantity of carbohydrate and its ability to raise the blood glucose.^4^

High GI and GL diets are quickly digested, absorbed and transformed into glucose. These processes accelerate insulin and glucose fluctuations, resulting in the early return of hunger, causing excessive caloric consumption. However, low GI and GL diets provide a slow and gradual insulin and glucose release in the bloodstream, thereby promoting increased fat oxidation, reducing lipogenesis and, consequently, increasing satiety and resulting in reduced food intake.^5^

Regarding these factors, some researchers have studied the association between the glycemic index and glycemic load and the risk of overweight in children.^6^^-^ 11 Nielsen et al.^10^ and Murakami et al.^11^ reported that high glycemic load and index diets increase the risk of overweight among children and adolescents; however, it has not been confirmed by other authors.^6^^-^ 9

Considering the effect of the glycemic index and glycemic load on hunger and high-energy intake, this study proposed to investigate the association between these parameters and the risk of overweight/obesity and high adiposity in children.

## Method

This cross-sectional study was nested in a cohort of live births between September 2004 and July 2005 in Diamantina, Brazil. The objective was to monitor growth and development in the first year of life.^12^ Details regarding the cohort selection and cross-sectional study have been described earlier elsewhere.^13^

The present study involved 5 year-old (±5 months) children of both genders from the cohort mentioned above.^12^ Data collection was done between July 2009 and July 2010. Each preschooler was visited in the respective home. Interviews and data collection commenced only after the parents signed the consent form permitting their child to participate in the study. The researchers were trained prior in data collection to avoid measurement errors. Four nutritionists collected the data. As a cross-sectional study nested in a cohort, the sample power was calculated pos-hoc using the parameter risk overweight/obese in relation to 3rd tertile for glycemic load adjusted for energy obtained by Poisson regression, which was 1.20. Using the statistical software “G*Power”, 85% power was obtained.

This study was approved by the Ethics Committee of the Federal University of the Valleys of Jequitinhonha and Mucuri-UFVJM, protocol number 039/08. Parents or guardians signed an informed consent form to participate in the study.

The nutritional status was assessed by body mass index-age (BMI/age) and adiposity was assessed by the triceps skinfold (TSF) and subscapular skinfold (SSF). Weight was measured using a portable, digital, electronic Kratos^®^ scale, with a maximum capacity of 150kg and 50g increments; height was measured on a portable stadiometer (Alturaexata^®^), with 0.1cm scale accuracy, according to Jellife^14^ recommendations. Skinfolds were measured using a compass check skinfold Lange^®^ Skinfold Caliper scale of up to 60mm with±1mm accuracy, on the right side of the body, based on the standardization of Lohman et al.^15^ Three measurements at each anatomical point were performed, and the average used in the analyses.

The z-score<−2 children identified with underweight, z-score≥−1 and z-score≤+1 normal weight, z-score>+1 and z-score≤+2 overweight, z-score>+2 obesity according to the BMI/age.^16^^,^ 17 The subjects were grouped into two categories for statistical analysis, underweight/normal weight were classified as normal weight and those with overweight/obesity as overweight. The z-score>+2.0 identified children with elevated adiposity (TSF/age, SBF/age).^16^ To identify the z-score of the children, the Software WHO Anthro and WHO Anthro plus versions 3.0.1 and 1.0.3, respectively, (WHO, Geneva). The two softwares were used because some children had already crossed 5 years of age when assessed.

The mothers of the children were also subjected to weight and height evaluations to obtain their body mass index. A BMI≥25kg/m^2^ was considered high.^18^

The data of both mothers and children were assessed on a single occasion, in the morning, utilizing the facilities of the Federal University of Jequitinhonha and Mucuri.

Children identified with some type of nutritional disorder received nutritional guidance but this was performed only after all the data were collected. These guidelines were directed at the children and their families.

The usual intake of preschool children was assessed by a Food-Frequency Quantitative Questionnaire (FFQQ) developed by Sales et al.^19^ adapted to suit this study after a pilot test. The parents or guardians filled in the FFQQ during the home visit. To increase the accuracy of determining the amount of food the children ingested, a photo album of food servings and eating utensils was used. To obtain the daily intake of each nutrient, we employed the methodology proposed by Willet^20^ ; the sum of the portion weights was multiplied by the frequency of consumption. The chemical composition of the food was determined using the Diet PRO software version 5i (Agromídia Software Ltda, Brazil).

To obtain the dietary glycemic index, the GI of a food was initially multiplied by its daily contribution to the carbohydrates available in each serving, after which, these products were added up, using the equation proposed by Wolever et al.^21^ The dietary glycemic load was obtained from the product of the dietary GI and the total available carbohydrate intake divided by 100.^21^ The available carbohydrate was obtained by subtracting the fiber content from the total carbohydrates available in the food.

The GI of each food was identified using the Table of Food Composition of the University of São Paulo (TBCA/USP)^22^ ; in the absence of this Table, we used the values listed in the International Table of Glycemic Index and Load glucose.^4^ When the food was not identified in any Table, the GI was determined by using the GI of a food with similar nutritional characteristics, following the identical order of reference. The food used as a standard was glucose, GI=100.

To better understand the relationship between dietary GI and GL and overweight/obesity and adiposity, these rates were adjusted by the dietary energy to monitor the effect of caloric intake in this relationship. Finally, to this we applied the residual nutrient method proposed by Willet et al.^23^ The residual nutrient intake of the nutrient was adjusted by the energy calculated by adding the residue of a linear regression model. The total energy intake was considered the independent variable and the absolute nutrient value of the dependent variable. The estimated energy requirement for each child was calculated by the formulas suggested by the Institute of Medicine (IOM).^24^

Considering that other variables, besides diet, can influence overweight/obesity and adiposity in preschoolers, we also decided to evaluate the socioeconomic status of the children (per capita family income in minimum wages), total breastfeeding period, maternal education (years of study) and overweight mothers. Parents or caregivers answered a structured questionnaire regarding these variables. The data on the breastfeeding practice was obtained prospectively from the cohort study, when the children were visited every month at home, thereby, reducing the possibility that the mother's memory bias could have occurred regarding the eating habits of their children. Tertile cutoffs were used to categorize the average meal glycemic load and glycemic index for subsequent statistical analyses. The relationship between the characteristics of the children and tertiles of dietary glycemic index and glycemic load was explored by ANOVA (parametric variables) and by Kruskal-Wallis test (nonparametric variables). The association between nutritional composition diets and nutritional status was assessed by *t*-test and Mann-Whitney U-test. Poisson regression was applied to determine the influence of the glycemic index and glycemic load on the condition of overweight in preschoolers. Initially, a bivariate analysis was done and the variables with *p*-value<0.20 were selected for multivariate analysis. The significance level was *p*<0.05.

All statistical analyses were performed using the Statistical Package for Social Sciences (SPSS) version 19.0 for Windows (SPSS Inc., Chicago, IL, USA). Ethical approval (ref. no. ETIC 545/08) was obtained from the Federal University of Minas Gerais.

## Results

The distribution of underweight, normal weight, overweight and obesity was 7 (3.0%), 185 (79.7%), 38 (16.4%) and 2 (0.9%), respectively. The prevalence of overweight/obesity was 17.3%, with 16.2% (n=23) in boys and 18.9% (n=17) in girls. High adiposity prevalence was 3.5% for the triceps skinfold and 6.9% for the subscapular skinfold.

Most of the children were males (61.2%), living with less than half of the minimum wage per capita (56.0%), were predominantly breastfed for less than three months (50.4%) and had mothers with normal BMIs (69.7%) who had less than nine years of education (50.4%).

Considering that meat, eggs and margarine have no carbohydrates and, therefore, do not contribute to the calculation of the IG and CG, only 87.7% of the foods that constitute the FFQQ contributed to glycemic diet profile. The remaining food was categorized and consumed by the children in the following frequencies: cereals and beans in 3-4×/week, milk/dairy products and sugar-added drinks in 2-3×/week, bakery products/biscuits, vegetables, fruits and sweets in 1-2×/week.

Table 1 shows the anthropometric, socioeconomic and dietary characteristics of the schoolchildren according to the tertiles of the dietary glycemic index. It was noted that BMI, TSF, and SSF children do not differ by the tertiles of GI. Based on the dietary variables, the average carbohydrate increased progressively among the tertiles, unlike the average fiber and protein intake, which decreased as the average GI increased among the tertiles.

**Table 1:** Anthropometric, socioeconomic and dietary characteristics of children in Diamantina, Brazil, according to the tertiles of dietary glycemic index.

Variables	Dietary glycemic index			
	1º Tertile (n=77)	2º Tertile (n=78)	3º Tertile (n=77)	*p*-value
BMI child (kg/m^2^, median±SD)	15.5 (1.8)	15.4 (1.6)	15.3 (1.5)	0.574^a^
Tricipital skinfold (mm, median±SD)	8.9 (3.3)	8.1 (2.4)	8.3 (2.7)	0.514^a^
Subscapular skinfold (mm, median±SD)	6.1 (2.5)	5.5 (1.9)	5.4 (1.7)	0.601^a^
Days of breastfeeding (median±SD)	271.2 (115.8)	254.9 (126.7)	267.2 (123.4)	0.596^a^
GL adjusted energy (%, mean±SD)	151.6 (45.9)	165.6 (35.4)	185.5 (34.2)	<0.001^b^
IG adjusted energy (%, median±SD)	64.0 (3.6)	66.8 (3.9)	70.7 (4.0)	<0.001^a^
Energy intake (g/day, mean±SD)	1528 (575)	1695 (641)	1528 (613)	0.146^b^
Carbohydrate intake^c^ (g/day, median±SD)	226.4 (45.2)	235.5 (24.9)	240.8 (25.6)	0.001^a^
Lipid intake^c^ (g/day, mean±SD)	63.3 (14.9)	61.1 (8.0)	59.1 (8.9)	0.058^b^
Protein intake^c^ (g/day, median±SD)	50.4 (12.9)	47.7 (9.1)	43.0 (8.4)	<0.001^a^
Fiber intake^c^ (g/day, median±SD)	17.1 (30.1)	12.0 (2.9)	11.6 (3.0)	<0.001^a^
Years maternal studied (median±SD)	10.0 (4.0)	8.6 (3.8)	7.4 (3.2)	<0.001^a^
Income per capita^d^ (median±SD)	422.5 (422.2)	269.1 (259.3)	207.2 (173.2)	<0.001^a^
BMI maternal (kg/m^2^, median±SD)	26.2 (11.5)	27.4 (14.5)	27.1 (13.7)	0.780^a^

BMI, body mass index; GL, glycemic load; IG, glycemic index; SD, standard deviation.

a Kruskal-Wallis test.

b ANOVA.

c Value adjusted for energy intake.

d Value related to the minimum wage in reals (R$510.00).

In the dietary GI analysis, no difference was observed between the anthropometric variables (BMI) and adiposity (TSF and SSF) and the tertiles of dietary GL.

Reviewing the behavior of the dietary variables distributed according to the GL tertiles, we discovered that while they resemble the GI regarding the carbohydrate and protein intake, they are not like that of fiber and energy. The energy consumption is significantly higher for the last GL tertile but with no difference for fiber intake (Table 2).

**Table 2:** Anthropometric, socioeconomic and dietary characteristics of children in Diamantina, Brazil, according to the tertiles of dietary glycemic load.

Variables	Dietary glycemic load			
	1º Tertile (n=77)	2º Tertile (n=78)	3º Tertile (n=77)	*p*-value
BMI child (kg/m^2^, median±SD)	15.3 (1.6)	15.2 (1.6)	15.7 (1.8)	0.163^a^
Tricipital skinfold (mm, median±SD)	8.5 (2.9)	8.4 (2.9)	8.5 (2.8)	0.978^a^
Subscapular skinfold (mm, median±SD)	5.8 (2.4)	5.5 (1.9)	5.6 (2.1)	0.888^a^
Days of breastfeeding period (median±SD)	282.3 (11.7)	262.3 (124.8)	248.6 (129.8)	0.637^a^
GL adjusted energy (%, mean±SD)	131.6 (33.8)	165.2 (6.4)	205.8 (34.0)	<0.001^b^
IG adjusted energy (%, median±SD)	64.4 (4.3)	67.3 (4.1)	69.8 (4.2)	<0.001^a^
Energy intake (g/day, mean±SD)	1531.6 (627)	1446 (534)	1779 (632)	0.002^b^
Carbohydrate intake^c^ (g/day, mean±SD)	218.9 (48.5)	234.3 (14.2)	249.5 (19.9)	<0.001^a^
Lipid intake^c^ (g/day, mean±SD)	62.8 (15.1)	61.8 (7.8)	58.9 (8.9)	0.092^b^
Protein intake^c^ (g/day, median±SD)	51.3 (13.6)	47.8 (6.7)	42.1 (8.7)	<0.001^a^
Fiber intake^c^ (g/day, median±SD)	15.2 (30.2)	12.7 (2.7)	12.89 (3.8)	0.677^a^
Years maternal studied (median±SD)	9.7 (4.0)	8.6 (3.7)	7.7 (3.6)	0.008^a^
Income per capita^d^ (median±SD)	374.0 (360.9)	280.1 (337.3)	244.6 (215.9)	0.013^a^
BMI maternal (kg/m^2^, median±SD)	26.4 (11.1)	27.1 (14.7)	27.8 (15.1)	0.835^a^

BMI, body mass index; GL, glycemic load; IG, glycemic index; SD, standard deviation.

a Kruskal-Wallis test.

b ANOVA.

c Value adjusted for energy intake.

d Value related to the minimum wage in reals (R$510.00).

Both analyses revealed that children from families with higher per capita income and having mothers with higher education levels ingested diets with better glycemic profiles (Tables 1 and 2).

The overweight group had a higher median carbohydrate intake (adjusted for energy) than the normal weight group, clearly indicating the effect of dietary composition on the nutritional status (Table 3).

**Table 3:** Nutritional composition diets according Body Mass Index.

Variables	Groups		
	Eutrophic (n=192)	Overweight (n=40)	*p*-value
GL adjusted energy (%, median±SD)	164.8 (38.9)	182.0 (37.1)	0.090^a^
IG adjusted energy (%, median±SD)	67.2 (4.6)	66.9 (5.3)	0.772^b^
Energy intake (g/day, median±SD)	1578 (599)	1517 (682)	0.742^b^
Carbohydrates intake^c^ (g/day, median±SD)	232.5 (35.2)	242.6 (22.8)	0.041^a^
Lipids intake^c^ (g/day, mean±SD)	61.5 (11.5)	59.5 (8.9)	0.226^b^
Protein intake^c^ (g/day, median±SD)	47.4 (11.2)	45.7 (7.9)	0.552^a^
Fiber intake^c^ (g/day, media±SD)	13.9 (19.3)	12.1 (2.2)	0.269^a^

GL, glycemic load; IG, glycemic index; SD, standard deviation.

a Student *t*-test.

b Mann-Whitney test.

c Value adjusted for energy intake.

The ratios of the crude and adjusted prevalence of overweight and adiposity by dietary glycemic load and glycemic index are shown in Table 4. Note that, after adjusting for the potential confounders (*e.g.*, maternal BMI, per capita income, exclusive breastfeeding, mother's education level), the dietary GI and GL were not independent risk factors for overweight/obesity and high adiposity in the children evaluated.

## Discussion

A reasonable number of studies have observed the influence of the quality of dietary carbohydrates on promoting weight gain and obesity.^25^^-^ 27 Low glycemic index and low glycemic load diets have been used to treat and prevent overweight and obesity in several studies, mainly in the United States.^28^ However, most of them were conducted on adults and elderly patients with chronic diseases. Against such a backdrop of scientific literature, the present work explores this hypothesis in children with 5 years of age.

On analyzing the upper tertile of dietary GI and GL, children were seen to consume a significantly higher carbohydrate level. This can be explained by the high frequency of consumption of foods that contribute towards increasing the GI and GL. The most frequently consumed food groups were cereals and beans, followed by sugary drinks. These foods are high in simple carbohydrates and, thus, contribute to an increased dietary GI. The fruits and vegetables that could decrease the GI in diets due to their high fiber content were consumed in a much lower frequency. It is noteworthy that the cereals consumed were all of the refined variety, because the FFQQ used did not include wholefoods. In this questionnaire design, wholefoods were not included as they were rarely consumed by the population studied.^19^ Besides, the regular consumption of wholefoods by children (5 years old) is very low, especially in this sample that included mostly the low income group.

The upper GI and GL tertiles have significantly lower protein levels and higher carbohydrate levels compared with the lower tertiles. Consequently, higher GL diets were composed of higher carbohydrate levels for the total caloric value, considering that the lipids do not differ significantly between the tertiles. Apart from this, in the univariate analysis, it was only the carbohydrate intake that differed between the groups, while overweight children consume diets with higher quantities of this nutrient. This was the reason for including the total carbohydrate intake in the statistical analysis as a confounding factor, avoiding the possible effect of dietary GI and GL for the risk of overweight and adiposity among the studied children.

In this work, the GI and GL were not associated with being overweight, as the average GI and GL did not differ between the normal weight and overweight groups. This finding may be related to the low prevalence of obese individuals (0.2%) found in this sample, when compared with the national prevalence (14.3%).^2^ Perhaps this association is best perceived in obese children and adults, the groups which generally consume larger food servings. Children 5 years of age normally consume small servings; therefore, even if they eat hyperglucidic foods, because of the small quantities, only lower GI and GL levels are observed. The methodology used to identify the GI and GL considers the amount of carbohydrate that the child consumes per serving,^21^ reaffirming the impact of serving size in the calculation of these parameters.

The association between glycemic diet profile and risk for being overweight in childhood is still not well established in the literature because of the limited number of studies in children and with quite controversial results.^6^^-^ 11

Scaglione et al.,^6^ evaluated the diet of 111 Italian children using the Food-Frequency Questionnaire (FFQ) and did not identify any association between body mass index and dietary GI and GL. A prospective study of 380 children, recruited from the Germanic Dortmund Nutrition and Anthropometric Longitudinally Designed Association, also found no such relationship.^7^ This hypothesis was also not confirmed in the study done among 316 children, 6-7 years of age, in Hong Kong, in which the dietary GL was obtained from three dietary recalls and overweight by BMI.^8^ Davis et al.,^9^ examined the BMI and high adiposity in 120 young overweight Latin-Americans by Dual Energy X-ray Absorptiometry (DEXA); the authors found that only total sugar intake, obtained by two dietary recalls, was associated with these parameters.

In contrast, other researchers have identified a positive association between the glycemic diet profile and overweight.^10^^,^ 11 Nielsen et al.^10^ studied a population of 485,364 Danish children and adolescents, using one dietary recall and the sum of skinfolds. They reported that the dietary GI and GL were positively associated with the parameters analyzed, only in the male adolescents. Murakami et al.^11^ evaluated Japanese children and adolescents in a longitudinal study in which the data were obtained from a semi-quantitative food consumption frequency questionnaire, self-reported by the students’ families; the authors also analyzed weight and height. Similar to the study of Nielsen et al.,^10^ these authors observed that the dietary GL was associated with an increased risk of overweight only among children and adolescent males.

Two studies found a positive association between overweight^10^ and high adiposity^11^ and GI and GL. In these studies, the overweight group had higher GI and GL averages when compared with the normal weight group, a finding not observed in our study. In addition, the sample size was likely to influence the statistical analysis. Murakami et al.^11^ and Nielsen et al.^10^ worked with much larger populations than the other studies.

The first four studies reported^6^^-^ 9 a variable that was included in the statistical analyses as a potential confounder, maternal overweight. This finding was in common with the present study. This same variable was not considered in the two studies^10^^,^ 11 which had identified a positive association between GI and GL and overweight. The fact is the maternal overweight is a potential confounding factor and warrants further analysis. Additionally, it is known that children with a genetic predisposition may be more vulnerable to the metabolic consequences of consuming foods with high GI and GL compared with those without a family history of overweight.^29^

The inconsistency in findings may be partially explained by the different characteristics presented in each study, such as the dietary habits and lifestyles of the populations studied, dietary assessment method employed, nutritional status measured and the potential confounders included (or not) in the analyses.

In this context, the present study includes some limitations. Among them, the cross-sectional design of the study may be inappropriate to investigate the frequency of food intake and anthropometric characteristics. In this case, the phenomenon of reverse causality may have occurred, as mothers of overweight children could have offered healthier food compared with the food they normally consumed. As the food frequency questionnaire^19^ addressed the dietary intake of the prior month and the interval between the invitation and data collection could exceed one month, these recent changes may have influenced the association that the study sought to investigate. This limited time may have been sensitive to the effect of reverse causality.

Another limitation is related to the GI Food Tables: In Brazil, we do not have a Food Table that includes a wide variety of local food. Thus, to study the GI, an international Table 4 had to be used, which could not accurately represent the intake of the children.

**Table 4:** Crude and adjusted prevalence ratios (PR) for overweight and adiposity according dietary glycemic load and glycemic index.

Variables	BMI			TSF			SSF	
	RP	*p*-value		RP	*p*-value		RP	*p*-value
**Crude analyse**								
*GL adjusted for energy intake*								
1º Tertile	1			1			1	
2º Tertile	0.82	0.623		0.99	0.987		0.82	0.738
3º Tertile	1.50	0.228		0.75	0.700		1.00	1.000

*GI adjusted for energy intake*								
1º Tertile	1			1			1	
2º Tertile	0.68	0.278		0.14	0.064		0.62	0.377
3º Tertile	0.81	0.538		0.43	0.027		0.50	0.241

**Adjusted analyse** ^a^								
*GL adjusted for energy intake*								
1º Tertile	1			1			1	
2º Tertile	0.77	0.509		1.12	0.872		0.94	0.917
3º Tertile	1.20	0.599		0.98	0.981		1.31	0.634

*GI adjusted for energy intake*								
1º Tertile	1			1			1	
2º Tertile	0.71	0.331		0.16	0.082		0.71	0.536
3º Tertile	0.81	0.579		0.57	0.465		0.61	0.441

GL, glycemic load; GI, glycemic index; BMI, body mass index; TSF, triceps skinfold; SS, subscapular skinfold.

a Adjusted analyses for body mass index maternal, income *per capita*, years maternal studied, carbohydrate intake adjusted for energy, all continuous variables. Analysis by Poisson regression.

In conclusion, dietary glycemic index and glycemic load are not independent risk factors for overweight/obesity and adiposity in 5 year-old Brazilian children. Despite these results, it is necessary to investigate this hypothesis in other studies, both prospective and observational, because the literature revealed that these indexes are strategic tools that can prevent and treat overweight. However, although that outcome is not established in childhood, teaching and directing children to select healthier, low-carbohydrate meals, should be a universal responsibility, especially within the family.
